# Serum 25(OH) vitamin d level is associated with treatment outcome of whole-body vibration (WBV) for osteopenia in girls with adolescent idiopathic scoliosis (AIS)

**DOI:** 10.1186/1748-7161-10-S1-O56

**Published:** 2015-01-19

**Authors:** Tsz Ping Lam, Bobby Kin Wah Ng, Fiona Wai Ping Yu, Echo Ka Ling Tsang, Wayne Yuk Wai Lee, Franco Tsz Fung Cheung, Huan Xiong Chen, Jack Chun Yiu Cheng

**Affiliations:** 1 Department of Orthopaedics and Traumatology, The Chinese University of Hong Kong, Hong Kong; 2 Joint Scoliosis Research Center of the Chinese University of Hong Kong and Nanjing University, China

## Objectives

AIS was associated with low bone mass which, apart from being an important health issue that could persist into adulthood, was also a significant prognostic factor for curve progression in AIS. We have performed a randomized controlled trial on WBV and reported its effect on increasing femoral neck areal bone mineral density (aBMD) mainly at the dominant leg but less obviously at the non-dominant side. The objective of this study was to evaluate the role of Vit-D in affecting the anabolic bone effect of WBV.

## Material and methods

This was a study nested within a randomized controlled trial with enrolment of 122 AIS girls (15-25 years old) with BMD Z-scores < -1. They were randomly allocated to the Treatment or Control group. The Treatment group received WBV by standing on a low-magnitude high-frequency WBV platform 20 mins/day, 5 days/week (acceleration 0.3g, frequency 35 Hz). The Control group received observation alone. The study period was one year. aBMD at bilateral femoral necks was measured with Dual-Energy X-ray Absorptiometry at baseline and at 12-month. Serum 25(OH)Vit-D level by liquid chromatography tandem mass spectrometry was measured at 6-month within the treatment period.

## Results

The mean age was 17.8(SD=1.5) years old and mean Cobb angle was 29.4(SD=8.8) degrees. Subgroup analysis for those with serum 25(OH)Vit-D>40nmol/L revealed not only the positive effects of WBV were greater at both sides, treatment effects were explicitly also noted at the non-dominant leg. In addition, the positive correlation between serum 25(OH)Vit-D and percentage increase in femoral neck aBMD that was not present in the Control group was explicitly detectable in the Treatment group at the non-dominant leg(p=0.004).

## Conclusions

The results strongly suggested the treatment effect of WBV could be enhanced through its synergistic factor interaction with Vit-D. The study carried significant clinical implication in that Vit-D insufficiency could affect negatively the treatment outcome of WBV for low bone mass in girls with AIS.

## Funding source

General Research Fund, Research Grants Council of Hong Kong (Project no: 467808 and 468809).

